# Genotype-Specific Differences in Phosphorus Efficiency of Potato (*Solanum tuberosum* L.)

**DOI:** 10.3389/fpls.2019.01029

**Published:** 2019-08-16

**Authors:** Katrin Wacker-Fester, Ralf Uptmoor, Verena Pfahler, Klaus J. Dehmer, Silvia Bachmann-Pfabe, Mareike Kavka

**Affiliations:** ^1^Department of Agronomy, University of Rostock, Rostock, Germany; ^2^Genebank Department, Satellite Collections North, Leibniz Institute of Plant Genetics and Crop Plant Research (IPK), Gross Luesewitz, Germany

**Keywords:** potato, phosphorus uptake, acid phosphatase, miR399, root system, tuber yield

## Abstract

Potato is considered to have a low phosphorus (P) efficiency compared to other crops. Therefore, P fertilization requirements are high. New cultivars with improved P efficiency may contribute to save limited mineral P sources and to reduce eutrophication of surface water bodies. The present study aims to characterize the P efficiency of different potato genotypes and to identify mechanisms that improve P efficiency in cultivated potato. A diversity set of 32 potato accessions was used to assess their P efficiency. From this set, five cultivars were selected and two pot experiments with different P-fertilization strategies including a non-fertilized control were conducted to estimate effects of P deficiency on general agronomic and P related traits, root development, phosphatase activity and micro RNA 399 (miR399) expression. Significant differences between the 32 genotypes were found for P utilization efficiency (PUtE). P acquisition efficiency (PAE) as P content in low P in relation to P content in high P was positively correlated to relative biomass production while PUtE was not. Selected genotypes displayed a strong relation between total root length and P content. Root phosphatase activity and miR399 expression increased under P deficiency. However, tuber yields of four cultivars, grown on a soil with suboptimal content of plant available P, were not significantly affected in comparison to yields of well-fertilized plots. We conclude from the present study that PUtE and PAE are important traits when selecting for plants requiring less fertilizer inputs but PAE might be more important for cropping on deficient soils. A large root system might be the most important trait for P acquisition on such soils and therefore in breeding for P efficient crops. Lowering P fertilizer inputs might not necessarily reduce tuber yields.

## Introduction

Phosphorus (P) is an essential macronutrient required for plant growth and metabolism and is applied as fertilizer in agricultural systems. Phosphorus is taken up from the soil as inorganic phosphate by phosphate/proton-co-transporters with dihydrogen phosphate as favored form (Nussaume et al., 2011). In most soils, P is not directly available for plants since it is immobilized rapidly and forms stable complexes with calcium, magnesium, aluminum, and iron cations and clay minerals (Lynch, 2007; López-Arredondo et al., 2014). The amount of P taken up by plants is a result of an interaction between dissolved P in the rhizosphere and plant’s requirements (Trehan, 2005). Even when mineral fertilizers are used, the concentration of available P in the soil solution can be lower than the level needed for optimal plant growth (Ma et al., 2009). Water-soluble P fractions of fertilizers (e.g., KH_2_PO_4_) are short-lived and most of the P ends up in more stable and less readily available P pools (Roberts and Johnston, 2015). Up to 90% of P in soils can be in organic form (Stutter et al., 2012). Plants developed different strategies to access those less available P forms. Organic acids, protons, and anions exuded from plant roots can release phosphate from stable complexes and increase the amount of plant available P (Hinsinger, 2001). Phosphatases, released by plant roots and microorganisms, are able to mobilize soil P *via* mineralization of organic P (Hinsinger et al., 2015). Phosphatases improve plant P acquisition from the soil but also increase internal inorganic phosphate concentrations if plant available P in the soil is limited. Therefore, both leaf and root phosphatase activity increase under P deficiency. A large group of purple acid phosphatases, which catalyze phosphate ester hydrolysis, have been described in *Arabidopsis* (Li et al., 2002), of which three are the major secreted and root-associated phosphatases under P deficiency (Wang et al., 2014). Twenty-two purple acid phosphatases were found in potato (Xie and Shang, 2018). The expressions of three potato purple acid phosphatases were characterized by Zimmermann et al. (2004), of which two responded to P deficiency with increased expressions in roots.

In terms of agricultural production, potato is the most important non-cereal food crop in the world (Wishart et al., 2013). Unfortunately, potato combines a high P-demand with low P uptake efficiency (Pursglove and Sanders, 1981; Dechassa et al., 2003) and thus needs high amounts of P. The P requirement of plants is highest during early development. Additionally to early growth, P is needed also for tuber production in potato. This P may originate from fertilizer or be translocated from the shoot (Houghland, 1960). Therefore, a sufficient P availability, resulting from appropriate fertilization strategies, is necessary to preserve plants from deficiency especially during this stage. However, even if P fertilization is carried out properly, plant roots acquire only 30% of the applied P. The rest becomes fixed by mineral soil compounds and microbes (Ha and Tran, 2014; López-Arredondo et al., 2014) or is lost *via* surface run-off or leaching where it contributes to eutrophication of surface water bodies (Correll, 1998; Smith and Schindler, 2009; Dhillon et al., 2017). To counteract this effect, further increasing governmental and environmental regulations concerning soil and water quality is being set into force. Thus, a rethinking of present agricultural fertilization practices is required in order to use limited P resources more efficiently (George et al., 2016) as globally decreasing rock P deposits cannot be easily substituted in fertilizer production (Ha and Tran, 2014; U.S. Geological Survey, 2017).

Apart from improved management practices aiming at the reduction of P losses to the environment and closing nutrient cycles, new cultivars with higher P efficiencies may contribute to a more sustainable use of P resources. In past years, advances were made towards an understanding of plant adaptations and reactions to P deficiency. Plants have developed different strategies to overcome P starvation and improve P acquisition efficiency (PAE) (Balemi and Schenk, 2009), which include modifications of root architecture and morphology and the release of phosphatases (Richardson et al., 2009; Lynch, 2011; Vandamme et al., 2016b; Parra-Londono et al., 2018). Several genes involved in signaling P deficiencies are known, for example, *PHOSPHATE STARVATION RESPONSE 1* and *PHOSPHATE 2* (Bari et al., 2006; Chiou and Lin, 2011). However, the genetic regulatory network resulting in an enhanced expression of genes encoding phosphatases in response to P starvation is to date not completely understood. Micro RNAs (miRNAs) play an essential role in P signaling and activation cascade, which potentially improve P efficiency of crops by enhancing P remobilization and acquisition. In particular, miR399 is highly expressed in P-starved tissues. In *Arabidopsis*, it is transported to the roots, where it silences *PHOSPHATE 2* (Lin et al., 2008). The resulting enhanced expression of phosphate transporters leads to an improved P-uptake capacity. In tomato, AtmiR399 overexpression additionally leads to increased phosphatase activity and proton release from roots, resulting in higher P acquisition from the soil (Gao et al., 2010). miR399 was found in potato (Zhang et al., 2013), but nothing is known about its role in P starvation responses in this crop to date.

Increasing knowledge about adaptation strategies could lead to the development of improved crop cultivars that maximize PAE and P utilization efficiency (PUtE), which is essential for enhanced food security and protection of water resources for future generations (López-Arredondo et al., 2014; Roberts and Johnston, 2015). Therefore, the objectives of the present study were to 1) characterize the P efficiencies of potato genotypes fertilized with high and low amounts of plant available P, 2) identify putative genotype specific mechanisms positively influencing PAE, e.g., by increasing root acid phosphatase activity and miRNA expression or adapting root morphology, and 3) evaluate the effect of P deficiency and different fertilization strategies on tuber yield in the field.

## Materials and Methods

### Pot Experiment With 32 Genotypes (Experiment A)

Thirty-two genotypes of cultivated potato (*Solanum tuberosum* L. subsp. *tuberosum*) adapted to Central and Western European growing conditions as well as non-adapted germplasm (*Solanum* spp.) from the Gross Luesewitz Potato Collections (GLKS) of the Leibniz Institute of Plant Genetics and Crop Plant Research (IPK) were characterized in a pot experiment with high and low amounts of plant available P. The cultivars within the diversity set were released between 1863 and 1995 and comprised maturity classes from early to very late (**Table 1**). Sprouted potato tubers (three biological replicates) were planted 3 cm deep in pots (21 cm high, 19.5 cm in diameter) filled with 6 kg of a soil–sand mixture (1:2). Soil from the control plots of a long-term field experiment, not fertilized with P since 1998 and located at the experimental station of the University of Rostock (see Experiment D), was used in this experiment. The soil was taken from a depth of 10 to 30 cm of the A horizon of a loamy sand (Haplic Luvisol). The soil–sand mixture had an initial pH value of 6.0 and a double-lactate soluble soil P content (P_dl_) of 15.5 mg kg^−1^, which is suboptimal for crop production according to the German soil P classification (Kape et al., 2019).

**Table 1:** Potato accessions with country of origin (country codes according to ISO 3166 ALPHA-3), year of release, maturity class, phosphorus acquisition efficiency (PAE), phosphorus utilization efficiency (PUtE), P concentration means (*n* = three biological replicates) and ANOVA *p* values for genotype, treatment, and G × T interaction.

Genotype	GLKS number	Origin	Release	Maturity	PAE	PUtE (g mg^−1^)		P conc. (mg g^−1^)	
					(%)	HP	LP	HP	LP
Gesa	11465	DEU	1984	interm.-late	93.3	0.42	0.46	2.38	2.17
Foremost	11450	GBR	1955	early	87.0	0.37	0.44	2.79	2.27
San	11828	POL	1980	late-very late	85.6	0.49	0.60	2.05	1.67
Abnaki	10093	USA	1970	late	84.4	0.55	0.57	1.83	1.75
Kijam	26465	CMR		—	77.3	0.53	0.68	1.88	1.48
Rema	11785	SVK	1983	intermediate	77.0	0.42	0.54	2.40	1.88
Amarilla olargada^a^	21389	CHL		—	76.1	0.51	0.60	1.97	1.72
Fransen	10873	NLD	<1893	intermediate	74.0	0.46	0.57	2.23	1.76
Fringilla	10891	DEU	1977	interm.-late	73.0	0.40	0.53	2.50	1.88
Zarewo	11982	UKR	1983	interm.-late	73.0	0.55	0.69	1.84	1.48
Amsel	10902	DEU	1956	early-interm.	72.6	0.50	0.56	2.02	1.81
Prince Edward Island Blue	12005	CAN		interm.-late	72.4	0.41	0.46	2.47	2.17
Pompadour (1992)	12227	FRA	1992	late	71.7	0.42	0.63	2.38	1.62
Linzer Speise	11598	AUT	1969	intermediate	71.6	0.44	0.62	2.30	1.63
Loshitskiy	11608	BLR	1962	late	70.7	0.45	0.63	2.22	1.59
Torva	11914	DNK	1987	early	67.6	0.46	0.63	2.17	1.63
Cara	12020	IRL	1973	very late	66.6	0.45	0.53	2.27	1.88
Hokkaiaka	11495	JPN	1965	late-very late	65.9	0.54	0.78	1.85	1.29
Russet Burbank	11817	USA	1917	very late	65.7	0.47	0.62	2.15	1.63
Fauna	11439	POL	1984	intermediate	65.1	0.38	0.53	2.63	1.91
Dobrin	11383	YUG	1962	interm.-late	64.9	0.36	0.53	2.78	1.91
Nepal 2	12216	NPL		late	62.7	0.44	0.51	2.29	1.97
Huinkul	26069	ARG	1946	—	62.4	0.49	0.61	2.10	1.65
Trogs Lichtblick	10351	DEU	1922	intermediate	59.9	0.47	0.66	2.15	1.52
Tarumae	11897	JPN	1969	intermediate	58.6	0.46	0.67	2.22	1.51
Dalat weiß	11368	VNM		late	57.7	0.51	0.72	1.98	1.39
Pinanza^b^	25513	PER		—	57.7	0.32	0.47	3.11	2.18
White Elephant	12034	USA	<1881	interm.-late	57.2	0.41	0.59	2.48	1.71
Ccompis^c^	25518	PER		—	54.2	0.38	0.64	2.70	1.55
Lady Christl	12405	NLD	1995	very early	51.8	0.48	0.68	2.09	1.47
Paterson’s Victoria	11723	GBR	1863	interm.-late	49.7	0.47	0.79	2.16	1.28
Weinberger Schloßkipfler	11968	AUT	1962	late	48.6	0.42	0.67	2.43	1.51
Mean					68.0	0.45	0.60	2.28	1.71
Standard deviation					10.9	0.06	0.09	0.30	0.26
Max					93.3	0.55	0.79	3.11	2.27
Min					48.6	0.32	0.44	1.83	1.28
*p*_Genotype_					ns	<0.001		<0.001	
*p*_Treatment_					na	<0.001		<0.001	
*p*_GxT_					na	0.005		ns	

^a^Native variety, ^b^S. juzepczukii Bukasov, native variety, ^c^S. tuberosum subsp. andigena Hawkes, native variety.

HP, high P supply; LP, low P supply; ns, not significant with p > 0.05, na: not applicable.

The potato genotypes were grown at two P levels: The soil was either amended with 333 mg P as KH_2_PO_4_ per kg soil–sand mixture (high P, HP) or no P was added (low P, LP). In both treatments, 417 mg N per kg were applied as NH_4_NO_3_ and 83 mg kg^−1^ Mg as MgSO_4_ × 7 H_2_O. Each pot was fertilized with 817 mg K per kg, either as KH_2_PO_4_ (HP) or KCl (LP). Fertilizers were divided into six doses, supplied at the beginning of the experiment and then every second week starting 3 weeks after planting. After 7 weeks, the following nutrients were added: 20 mg kg^−1^ Ca, 10 mg kg^−1^ S, 0.09 mg kg^−1^ B, 0.35 mg kg^−1^ Fe, 0.055 mg kg^−1^ Mn, 0.068 mg kg^−1^ Zn, 0.0017 mg kg^−1^ Cu, and 0.0083 mg kg^−1^ Mo. From week 4 to week 11, *Phytophtora infestans* was controlled by weekly sprayings using Shirlan^®^ (Syngenta Agro, Maintal, Germany) and Acrobat^®^ plus (BASF SE, Ludwigshafen, Germany) alternately. The experimental design was a randomized complete block design with three replications. The experiment was carried out outside in a large wire cage to prevent large animals and litter from entering the area (**Figure S1**). Plants were watered with tap water according to plants’ requirements. Percolated water was collected in a cup below the pots and replenished. Shoots were harvested 80 or 90 days after planting according to genotype-specific development stages. Tubers were harvested 109 days after planting and fresh matter was determined. Tubers were sliced (2 cm) and both shoots and tubers were dried for 3 days at 60°C before dry matter and P concentration were determined.

### Pot Experiment With Tubers (Experiment B)

Potato tubers (about 35 mm in size) of the genotypes Amsel, Gesa, Paterson’s Victoria, and Weinberger Schloßkipfler were planted 3 cm deep in pots filled with 6 kg of a soil–sand mixture (1:3) with three different P fertilizer treatments. As in Experiment A, the soil was taken from a depth of 10 to 30 cm of a loamy sand at the experimental station of the University of Rostock, Germany. Since a different soil as in Experiment A was used, the soil–sand mixture had an initial pH value of 5.1 and a P_dl_ of 18.7 mg kg^−1^, which again is a suboptimal P status according to the German soil P classification (Kape et al., 2019). The soil was either amended with 30 mg P per kg soil–sand mixture as KH_2_PO_4_ (HP), 30 mg P per kg as C_6_H_16_CaO_24_P_6_ (calcium phytate, Sigma Aldrich, Taufkirchen, Germany, organic P, OP), or no P was added (LP). Additionally, HP-fertilized pots were amended with additional 90 mg K per kg as KCl, and OP- and LP-fertilized pots were amended with 167 mg K per kg as KCl. For the HP and LP treatments, 20 mg kg^−1^ Ca were added as CaCl_2_, while 13 mg kg^−1^ Ca as CaCl_2_ were added to attain the same fertilization level in OP. In all treatments, 83 mg N per kg were applied as NH_4_NO_3_, 17 mg kg^−1^ Mg as MgSO_4_ × 7 H_2_O as well as 0.083 mg kg^−1^ B as H_3_BO_3_, 0.333 mg kg^−1^ Fe as FeSO_4_ × 7 H_2_O, 0.05 mg kg^−1^ Mn as MnSO_4_ × H_2_O, 0.067 mg kg^−1^ Zn as ZnSO_4_ × 7 H_2_O, 0.0017 mg kg^−1^ Cu as CuSO_4_ × 5 H_2_O, and 0.0083 mg kg^−1^ Mo as Na_2_MoO_4_ × 2 H_2_O. Phosphorus was added in one dose at the beginning of the experiment end of April 2017. K, N, and Ca were divided into two doses and supplied at the beginning of the experiment and 3 weeks after planting. All other nutrients were supplied 3 weeks after planting. The experimental design was completely randomized. The pots and seven control pots per treatment without plants were placed in a greenhouse for a cultivation period of 8 weeks and watered with tap water according to plants’ needs. Percolated water was collected in a cup below the pots and replenished. Between three and nine plants per genotype and fertilizer level were harvested 8 weeks after planting end of June 2017. Soil samples from the rhizosphere were taken to determine acid phosphatase activity. To collect these samples, plants were removed from the pots and adherent soil was gently brushed from the roots to small sampling bags. The samples were stored at −20°C until analysis. Plants were divided into shoot, root, and—if present—tubers. Images of fresh roots were acquired using a scanner (CanoScan LiDE 120, Canon, Krefeld, Germany) with a resolution of 300 dpi. A black background maximized the contrast. Image processing to determine total root length and specific root length (as total root length divided by volume of the root itself) was carried out with GiA Roots (Galkovskyi et al., 2012). Dry matter and P concentration was determined after drying the plant samples for 5 days at 60°C.

### Pot Experiment With *In Vitro* Plants (Experiment C)

*In vitro* propagated potato plantlets of the genotypes Amsel and Russet Burbank were planted in 3-L pots filled with autoclaved sand, in a way that about one third of the shoot was belowground, and grown in a greenhouse at 20°C and 12 h additional light when natural light was below 5 klux. Plants were irrigated every second day with a nutrient solution comprising 5 mM KNO_3_, 5 mM Ca(NO_3_)_2_ × 4 H_2_O, 2 mM MgSO_4_ × 7 H_2_O, 0.1 mM FeSO_4_ × 7 H_2_O, 50.3 µM KCl, 25.07 µM H_3_BO_3_, 2.01 µM MnSO_4_ × H_2_O, 2.02 µM ZnSO_4_ × 7 H_2_O, 0.52 µM CuSO_4_ × 5 H_2_O, and 0.50 µM Na_2_MoO_4_ × 2 H_2_O with pH 5.8. Phosphorus was added as KH_2_PO_4_ (0.5 mM) in the high-P treatment. In the low-P treatment, KCl was used in an equimolar concentration. Pots were flooded with distilled water once per week to avoid accumulation of nutrients. Plant height was measured from the sand surface to the youngest leaf (> 2 cm length). Four plants per sampling date (1, 2, 3, and 6 weeks after planting) and treatment were destructively harvested and divided into leaves, shoot axis above- and belowground, roots, and—if present—tubers. An approximately 3-cm-long, representative root part was stored in the respective nutrient solution used for irrigation (maximum 2 h) until acid phosphatase activity was measured in weeks 2, 3, and 6. The remaining roots were in total (weeks 1 to 3) or partly (final harvest) frozen in liquid nitrogen and stored at −80°C until RNA extraction. At final harvest, a part of the roots was used to measure dry matter and P concentration. Both dry matter and P concentration were determined after drying samples for 1 week at 60°C.

### Field Experiment (Experiment D)

The field trial was conducted in 2016 at the experimental station of the University of Rostock within the plots of a long-term field experiment established in 1998 (Zicker et al., 2018). The long-term field experiment is arranged as a randomized split-plot design assigning organic fertilizer supply (no organic fertilizer, cattle manure or compost) to main plots and inorganic fertilizer supply [no P fertilizer, triple-superphosphate (TSP), or biomass ash] to subplots, which results in nine treatments with only organic (2), only inorganic (2), combined organic/inorganic P fertilization (4), and a control without P fertilization. Each treatment has four replicates. We used those plots with only cattle manure or TSP as P fertilizer (in total 409 and 391 kg P ha^−1^ applied from 1998 to 2016; Zicker et al., 2018) and the control with no P supply. In spring 2016, the P_dl_ content of the control, TSP, and cattle manure plots were on average 27.3, 33.2, and 36.3 mg P kg^−1^ soil (0–30 cm depth). Within each plot, eight rows of potatoes were planted with a row distance of 75 cm and a within-row planting distance of 30 cm in April. In the four midrows, six pre-sprouted tubers of the genotypes Amsel, Paterson’s Victoria, and Weinberger Schloßkipfler and five of the genotype Gesa were planted. The four other rows and the borders of each row were filled with cv. Gala to avoid border row effects. At harvest in August, tuber fresh matter of each genotype and plot was determined and yield was calculated.

### Plant and Soil P Measurements

Dried plant samples were ground, dried at 105°C for 4 h, and burnt at 550°C in a muffle furnace for 5 h. Total P was extracted in hydrochloric acid (25%) according to Page et al. (1982). Phosphorus concentrations were determined by inductively coupled plasma-optical emission spectroscopy (ICP-OES, Optima 8300, Perkin Elmer, USA) at a wavelength of 214 nm. For Experiment A, P concentrations were determined using the vanadate-molybdate method according to Gericke and Kurmies (1952) and photometrical measurements at a wavelength of 430 nm (Spekol 11, Carl Zeiss Jena, Jena, Germany).

PAE was calculated from the ratio between P content (mg plant^−1^) in LP and HP times 100 after López-Arredondo et al. (2014) and gives a percentage value for the responsiveness to P scarcity (small percentages indicate higher differences in P uptake between LP and HP). In Experiment A, plants from the same block were used as one pair. Phosphorus utilization efficiency (PUtE; g mg^−1^) was calculated as dry matter (g plant^−1^) over P content (mg plant^−1^).

Plant-available P was analyzed from air-dried and sieved (< 2 mm) soil samples using a modified double-lactate solution method according to Riehm (1948). Briefly, P_dl_ was extracted from 12 g soil by shaking overhead for 90 min at 35 rotations per minute with 150 ml of a calcium lactate (0.4 M C_6_H_10_CaO_6_ × 5 H_2_O) and hydrochloric acid (0.5 M HCl) solution at pH 3.6. Phosphorus concentration of the double-lactate solution was determined by ICP-OES (Optima 8300, Perkin Elmer, USA) at a wavelength of 214 nm. For Experiment A, P concentrations were measured using the vanadate-molybdate method and photometrical measurements at a wavelength of 430 nm (Specord 40, Analytic Jena, Jena, Germany). Soil pH was measured electrochemically after extraction with CaCl_2_ (0.01 M; 10 g soil with 25 ml solution).

### Acid Phosphatase Measurements

Rhizosphere phosphomonoesterase activity in Experiment B was analyzed according to Tabatabai and Bremner (1969) as follows: One gram of fresh soil was weighed into 15-ml tubes and incubated for 1 h at 37°C with 4 ml of modified universal buffer (pH 6.5) and 1 ml of 25 mM para-nitrophenyl phosphate (pNPP) as analogue phosphate monoester substrate. The reaction was stopped by adding 1 ml of 0.5 M CaCl_2_ and 4 ml of 0.5 M NaOH. The samples were mixed and filtered. One milliliter of the filtrate was diluted with 5 ml of deionized water and measured photometrically at 400 nm (Specord 40, Analytic Jena, Jena, Germany).

For measurements of root-associated acid phosphatase activity in Experiment C (four biological replicates), root parts were washed in a sodium-acetate buffer (50 mM, pH 5.5) and carefully patted dry with paper towels. They were shaken for 1 h in 4.5 ml of sodium-acetate buffer (50 mM, pH 5.5) with 600 µl pNPP (0.1%) at room temperature. The reaction was stopped by adding 0.9 ml of NaOH (0.4 M). Para-nitrophenol (pNP) in the solution was measured photometrically at 410 nm (Specord 40, Analytic Jena, Jena, Germany) with three technical replicates. The mean value of the technical replicates was used for further calculations. Fresh matter of root parts was determined. Phosphatase activity was calculated as formed pNP per gram of root and hour against a standard curve with pNP.

### miRNA Expression Measurement

Micro RNA expression was measured using stem–loop primers after Chen et al. (2005) and Kramer (2011). Total RNA was extracted from 50 to 100 mg of frozen root tissue (Experiment C, three biological replicates). Root material was homogenized in 1 ml of peqGold TriFast^™^ (Peqlab, VWR, Darmstadt, Germany) according to the manufacturer’s instructions using Precellys^®^ Evolution tissue homogenizer (Bertin Technologies, Montigny-le-Bretonneux, France). Total RNA was quantified by Nanodrop measurement (Thermo Fisher Scientific, Waltham, USA), and integrity was tested by agarose gel electrophoresis. To remove genomic DNA, 1 µg of RNA was treated with DNase I, RNase-free (Thermo Fisher Scientific, Waltham, USA). cDNA was transcribed from 0.4 µg of RNA with the RevertAid H Minus First Strand cDNA Synthesis Kit (Thermo Fisher Scientific, Waltham, MA, USA) using 4 pmol of the stem–loop primers 5′-GTCGTATCCAGTGCAGGGTCCGAGGTATTCGCACTGGATACGACCAGGGC-3′ and 5′-GTCGTATCCAGTGCAGGGTCCGAGGTATTCGCACTGGATACGACTGTTTG-3′ (after Chen et al., 2005) and 20 pmol oligo(dT)-primers per reaction (in total 20 µl) in a primer mix.

Quantitative real-time PCR (qRT-PCR) for mature miRNA 399a-f [3p-stem, according to miRBase (Kozomara and Griffiths-Jones, 2014), from now on miR399] and *EF1-α* (NM_001288491 in NCBI database), coding for an elongation factor and used as reference gene, was performed with two technical replicates (mean value was used for further calculations) in 96-well plates using a CFX96™ Real-Time PCR System (BioRad Laboratories, Hercules, USA). The reaction volume (20 µl) consisted of 10 µl of Maxima SYBR Green qPCR Master Mix (Thermo Fisher Scientific, Waltham, USA), 0.6 µl of each forward and reverse primer (10 µM, for *EF1-α*: 5′-CACTTCCCACATTGCTGTAAAG-3′ and 5′-CTTCAAGAACTTAGGCTCCTTC-3′, for miR399: 5′-AGAGGTGCCAAAGGAGAGC-3′ and 5′-CAGTGCAGGGTCCGAGG-3′), 3.8 µl of nuclease-free diethyl pyrocarbonate-treated water (Carl Roth, Karlsruhe, Germany), and 5 µl of the cDNA solution (1:10 diluted). The program consisted of an initial denaturation at 95°C (3 min), followed by 40 (for *EF1-α*) or 45 (for miR399) cycles at 95°C for 10 s, 57°C for 30 s, and 72°C for 60 s. Final melting curve analyses from 57°C to 95°C with 57°C for 30 s and a 0.5°C per 5 s increasing temperature gradient were used to check primer specificity. To calculate primer efficiency, raw data were loaded into LinRegPCR version 2017.0 (Ruijter et al., 2009). The mean efficiency for each primer pair was calculated over all samples per gene after baseline subtraction. Cq values were determined using a fluorescence threshold of 180.3. Relative expression values for each sample were calculated against *EF1-α* as reference gene.

### Statistical Analyses

R (version 3.3.2, R Core Team, 2016) was used for all statistical tests. Prior to carrying out two-way analysis of variance (ANOVA) and Tukey’s HSD test, residuals of all data sets were checked visually for normal distribution and homogeneity of variances. Based on the results, PAE data of Experiment A was arcsine square root transformed and expression data of Experiment C were log transformed before further analyzing the data. Pearson’s correlation coefficients and *p* values of correlations were calculated between all traits measured in Experiment A (means of each genotype) and Experiment B. Linear regression analysis was carried out on the relation between total root length and P content in the different treatments of Experiments B. For field data of Experiment D, a linear mixed effects model implemented in R package “lme4” (Pinheiro et al., 2012) with genotype and fertilizer treatments as fixed effects and replications as random effect was used and significant factor and interaction effects were calculated with analysis of deviance from R package “lmerTest” (Kuznetsova et al., 2016). Multiple comparisons using “glht” and Tukey’s test in the R package “multcomp” (Hothorn et al., 2008) were conducted and differences of genotype and treatment means were considered to be significant if *p* ≤ 0.05.

## Results

### Phenotype Screen in Contrasting P Environments (Experiment A)

**Figure 1:**
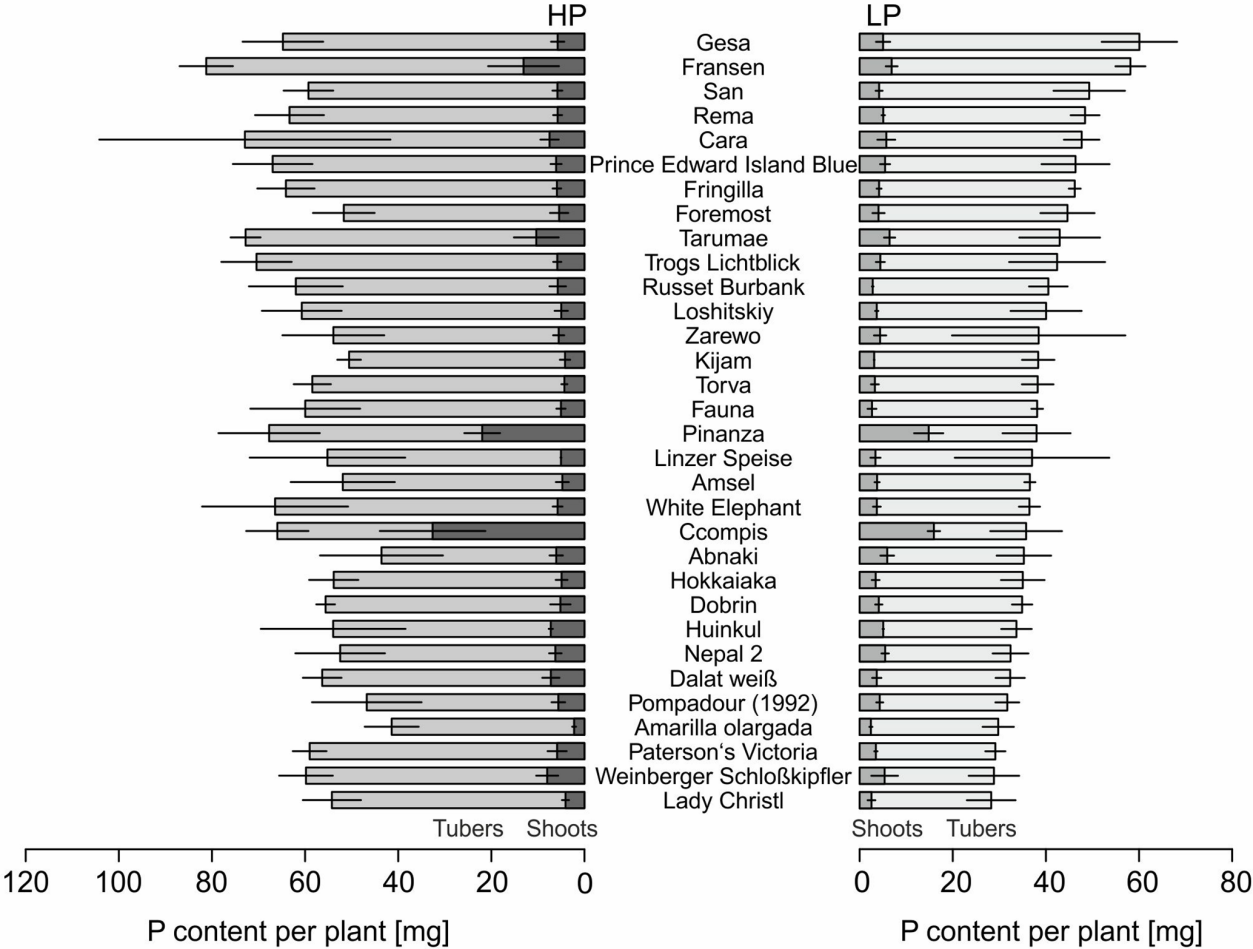
Means and standard deviations (*n* = three biological replicates) of phosphorus contents in shoots and tubers of potato plants grown with phosphate (HP) or without additional phosphate supply (LP) in a low P soil–sand mixture for about 12 weeks. Accessions are sorted by P content in LP.

**Figure 2:**
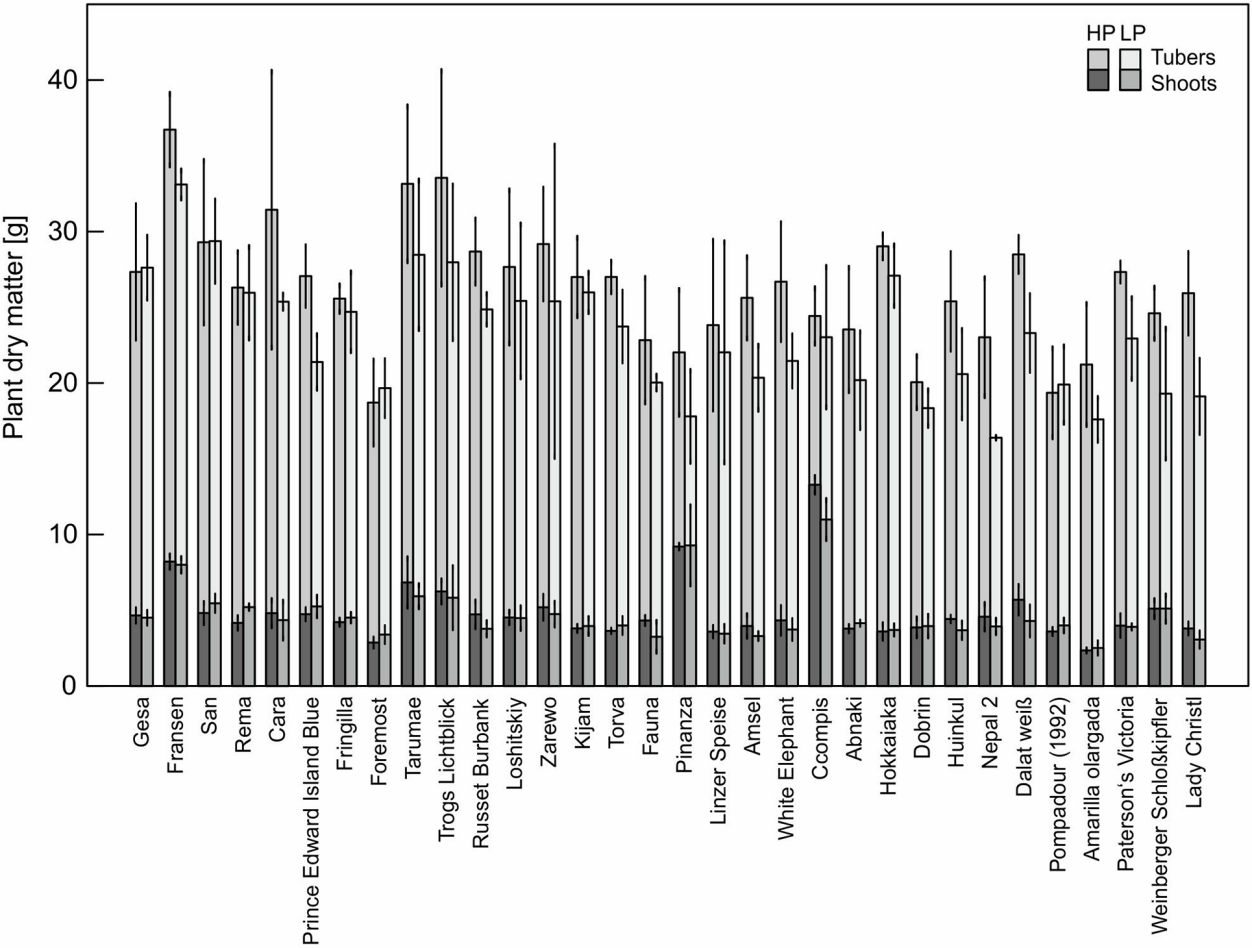
Means and standard deviations (*n* = three biological replicates) of shoot and tuber dry matter of potato plants grown with phosphate (HP) or without additional phosphate supply (LP) in a low P soil–sand mixture for about 12 weeks. As in **Figure 1**, accessions are sorted by P content in LP.

The P content of 32 potato genotypes grown in HP ranged between 41 and 81 mg plant^−1^ for Amarilla olargada and Fransen, respectively, and was significantly higher (*p* < 0.001) than under LP conditions, where Lady Christl had 28 and Gesa 60 mg P plant^−1^ (**Figure 1**). Differences between genotypes were statistically significant (*p* < 0.001), while the genotype × P level interaction effect was not significant (*p* > 0.05). Tuber P content was on average 87.9% (HP) and 86.8% (LP) of total plant P (**Figure 1**). Exceptions were the two Peruvian genotypes Ccompis (51 and 54% in HP and LP) and Pinanza (69 and 60% in HP and LP) due to their high shoot biomass (**Figure 2**). Total dry matter was significantly smaller in LP (*p* < 0.001) and different between genotypes (*p* < 0.001), while the genotype × P level interaction effect was not significant (*p* > 0.05).

PAE, the ratio of P content in LP to P content in HP, ranged from 93% for Gesa to 48% for Weinberger Schloßkipfler (**Table 1**). PAE was not related to maturity class or origin. PUtE was for all genotypes significantly higher under LP conditions (*p* < 0.001) and differed significantly between genotypes (*p* < 0.001). PUtE in LP was highest for Paterson’s Victoria (0.79 g mg^−1^) and lowest for Foremost (0.44 g mg^−1^). Highest PUtE in HP was measured for Abnaki and Zarewo (0.55 g mg^−1^), while Pinanza had the lowest PUtE (0.32 g mg^−1^). Phosphorus concentrations ranged between 1.83 mg g^−1^ and 3.11 mg g^−1^ in HP and 1.28 mg g^−1^ and 2.27 mg g^−1^ in LP (**Table 1**).

**Figure 3:**
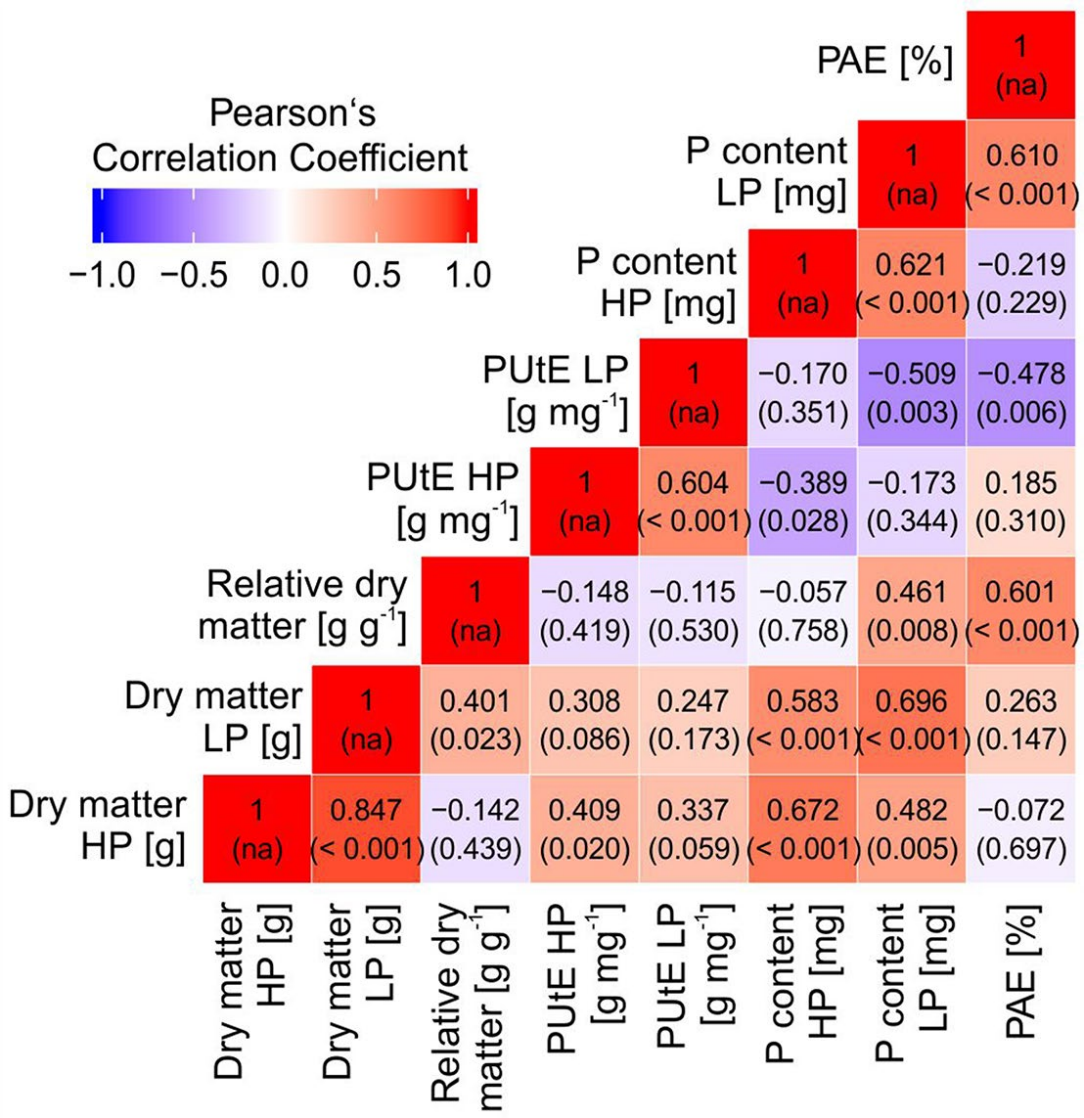
Pearson’s correlation coefficients (and *p* values of correlation) between dry matter, relative dry matter, phosphorus utilization efficiency (PUtE), plant phosphorus content at high (HP) and low P (LP), and phosphorus acquisition efficiency (PAE).

Dry matter was significantly correlated with PUtE in HP and with plant P content in HP and LP (**Figure 3**). PAE was significantly correlated with relative plant dry matter, the ratio between dry matter in LP and HP. A strong negative correlation was observed between PAE and PUtE in LP.

In order to identify genetic, physiological, or morphological mechanisms involved in P efficiency, we selected genotypes, suitable for cultivation under Central European climate conditions, with high (Gesa), intermediate (Amsel and Russet Burbank), and low (Weinberger Schloßkipfler and Paterson’s Victoria) PAE (**Table 1**) for further analyses although genotypic differences in PAE were statistically not significant.

### Influence of P Fertilizer on P and Growth Traits and Rhizosphere Phosphatase Activity (Experiment B)

While there were no significant treatment effects (phosphate, phytate, or no P fertilizer) on plant dry matter (**Table 2**), differences between genotypes grown from tubers were statistically significant (*p* < 0.05). Weinberger Schloßkipfler had the lowest dry matter across all treatments (9.70 g) while it was highest for Paterson’s Victoria (12.2 g). Phosphorus content was lowest for plants without additional P supply and differed significantly from the P content of fertilized plants (*p* < 0.001, **Table 2**). The genotype × treatment interaction effect was significant as well (*p* < 0.05). Amsel, Paterson’s Victoria, and Weinberger Schloßkipfler had highest P contents when mineral P fertilizer was used while Gesa had the highest P content after organic P supply. PUtE and plant P concentrations differed significantly between treatments (*p* < 0.001, **Table 2**). Similar to Experiment A, higher values were found under LP and OP than under HP. PUtE correlated negatively and significantly with P content across treatments (*r* = −0.3, *p* = 0.008, **Figure S2**).

**Table 2:** Means for traits of potato cultivars grown in the different phosphorus fertilization regimes HP (KH_2_PO_4_), OP (C_6_H_16_CaO_24_P_6_), and LP (without P supply).

Trait/cultivar	HP		OP		LP		Mean	
Plant dry matter (g plant^−1^)								
Amsel	11.3		11.7		12.6		*11.9*	*AB*
Gesa	9.8		13.5		10.8		*11.4*	*AB*
Paterson’s Victoria	13.8		12.0		12.0		*12.6*	*B*
Weinberger Schloßkipfler	9.7		10.9		8.63		*9.8*	*A*
*Mean*	*11.2*	*ns*	*12.0*		*11.0*			
Root/shoot ratio (g g^−1^)								
Amsel	0.51		0.50		0.46		*0.49*	ns
Gesa	0.51		0.42		0.48		*0.47*	
Paterson’s Victoria	0.41		0.44		0.48		*0.44*	
Weinberger Schloßkipfler	0.54		0.55		0.57		*0.55*	
*Mean*	*0.49*	*ns*	*0.48*		*0.50*			
Plant P content (mg plant^−1^)								
Amsel	41.5		32.4		32.4		*35.4*	*ns*
Gesa	34.8	ab	45.4	b	29.1	a	*36.4*	
Paterson’s Victoria	49.6	b	31.6	a	34.4	a	*38.5*	
Weinberger Schloßkipfler	37.7		35.5		27.3		*33.5*	
*Mean*	*40.9*	*b*	*36.2*	*b*	*30.8*	*a*		
Plant P concentration (mg g^−1^)								
Amsel	3.70		2.91		2.56		*3.11*	*ns*
Gesa	3.70		3.32		2.75		*3.21*	
Paterson’s Victoria	3.62		2.63		2.98		*3.16*	
Weinberger Schloßkipfler	4.19		3.41		3.27		*3.56*	
*Mean*	*3.80*	*b*	*3.15*	*a*	*2.93*	*a*		
P utilization efficiency (g mg^−1^)								
Amsel	0.27		0.36		0.40		*0.34*	*ns*
Gesa	0.28		0.31		0.37		*0.32*	
Paterson’s Victoria	0.28		0.38		0.35		*0.33*	
Weinberger Schloßkipfler	0.25		0.31		0.32		*0.29*	
*Mean*	*0.27*	*a*	*0.34*	*b*	*0.36*	*b*		
Rhizosphere acid phosphatase activity (µg pNP g^−1^ h^−1^)								
Amsel	21.6		18.4		15.8		*18.6*	*B*
Gesa	14.9		18.6		18.3		*17.3*	*B*
Paterson’s Victoria	13.5		6.08		15.0		*11.5*	*AB*
Weinberger Schloßkipfler	13.7		12.1		17.7		*14.5*	*B*
Without plant	5.2		6.3		7.5		*6.4*	*A*
* Accession mean*	*15.9*	*ns*	*13.8*		*16.7*			
Total root length (cm)								
Amsel	3577		3068		2663		*3103*	*AB*
Gesa	2701		3213		3401		*3105*	*AB*
Paterson’s Victoria	3975		3926		3356		*3752*	*B*
Weinberger Schloßkipfler	2814		3048		2493		*2785*	*A*
*Mean*	*3267*	*ns*	*3314*		*2978*			
Specific root length (cm cm^−3^)								
Amsel	1612	a	1680	a	1837	b	*1710*	*B*
Gesa	1689		1554		1584		*1609*	*AB*
Paterson’s Victoria	1627	ab	1559	a	1736	b	*1641*	*AB*
Weinberger Schloßkipfler	1480	a	1527	a	1707	b	*1571*	*A*
*Mean*	*1602*	*a*	*1580*	*a*	*1716*	*b*		

Different uppercase letters indicate statistical significance between cultivar overall means, different lowercase letters indicate statistical significance between treatments according to Tukey’s HSD (p < 0.05). If G *×* T interaction was significant according to ANOVA (p < 0.05), means separation was carried out between treatments for each cultivar separately. ns: not significant.

Cultivar and treatment overall means are displayed in italics.

**Figure 4:**
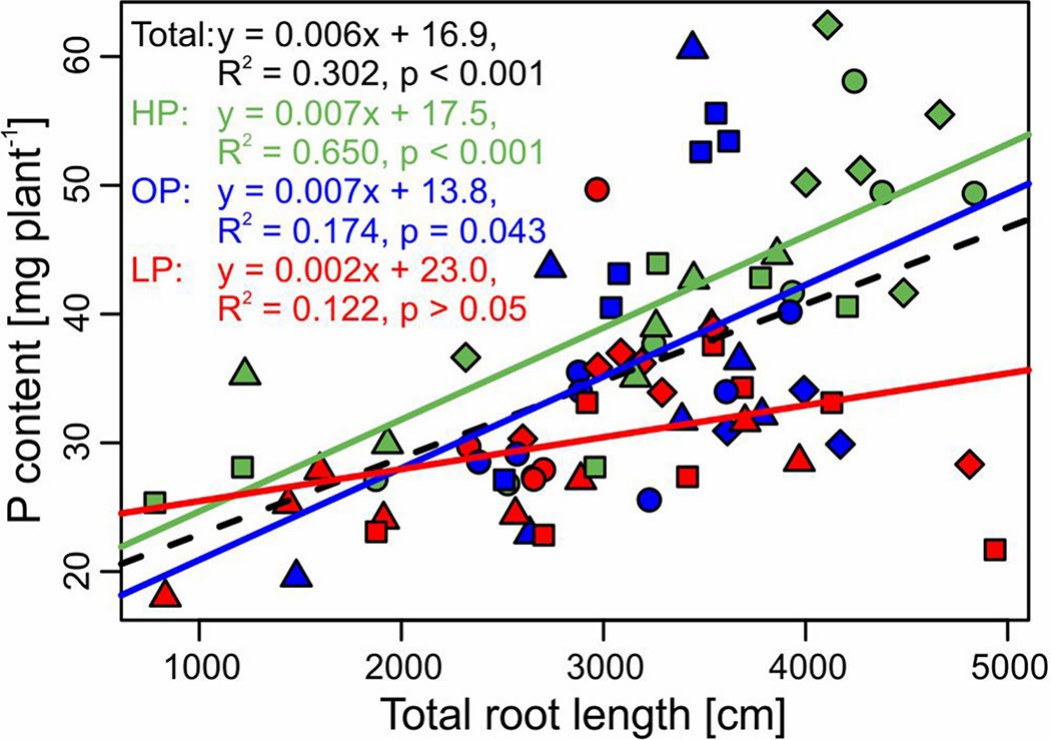
Relationship (linear regression) between P content and total root length of the potato cultivars Amsel (circle), Gesa (square), Paterson’s Victoria (diamond), and Weinberger Schloßkipfler (triangle) fertilized with KH_2_PO_4_ (HP, green), C_6_H_16_CaO_24_P_6_ (OP, blue), or without P supply (LP, red) and across all fertilizers (black, dashed line). Regression equations, multiple *R* ^2^ and *p* values are given. Individual symbols are individual plants.

While treatments were statistically not different for both root/shoot ratio and total root length, specific root length increased significantly in the LP treatment (*p* < 0.001, **Table 2**). Across treatments, P content correlated significantly negative with root/shoot ratio (*r* = −0.32, *p* = 0.004) and specific root length (*r* = −0.32, *p* = 0.004, **Figure S2**). Among root traits, the strongest correlation was found between P content and total root length (*r* = 0.55, *p* < 0.001). The regressions between P content and total root length for the different treatments and across treatments are displayed in **Figure 4**.

Rhizosphere acid phosphatase activity was statistically not different between both genotypes and treatments, but significantly higher than in control pots without plants (**Table 2**). Rhizosphere acid phosphatase activity ranged between 6.08 µg pNP g^−1^ h^−1^ for Paterson’s Victoria using organic P and 21.6 µg pNP g^−1^ h^−1^ for Amsel with mineral P supply. Correlation between P content and rhizosphere acid phosphatase activity was statistically not significant (**Figure S2**).

### Root Phosphatase Activity and miR399 Expression (Experiment C)

**Figure 5:**
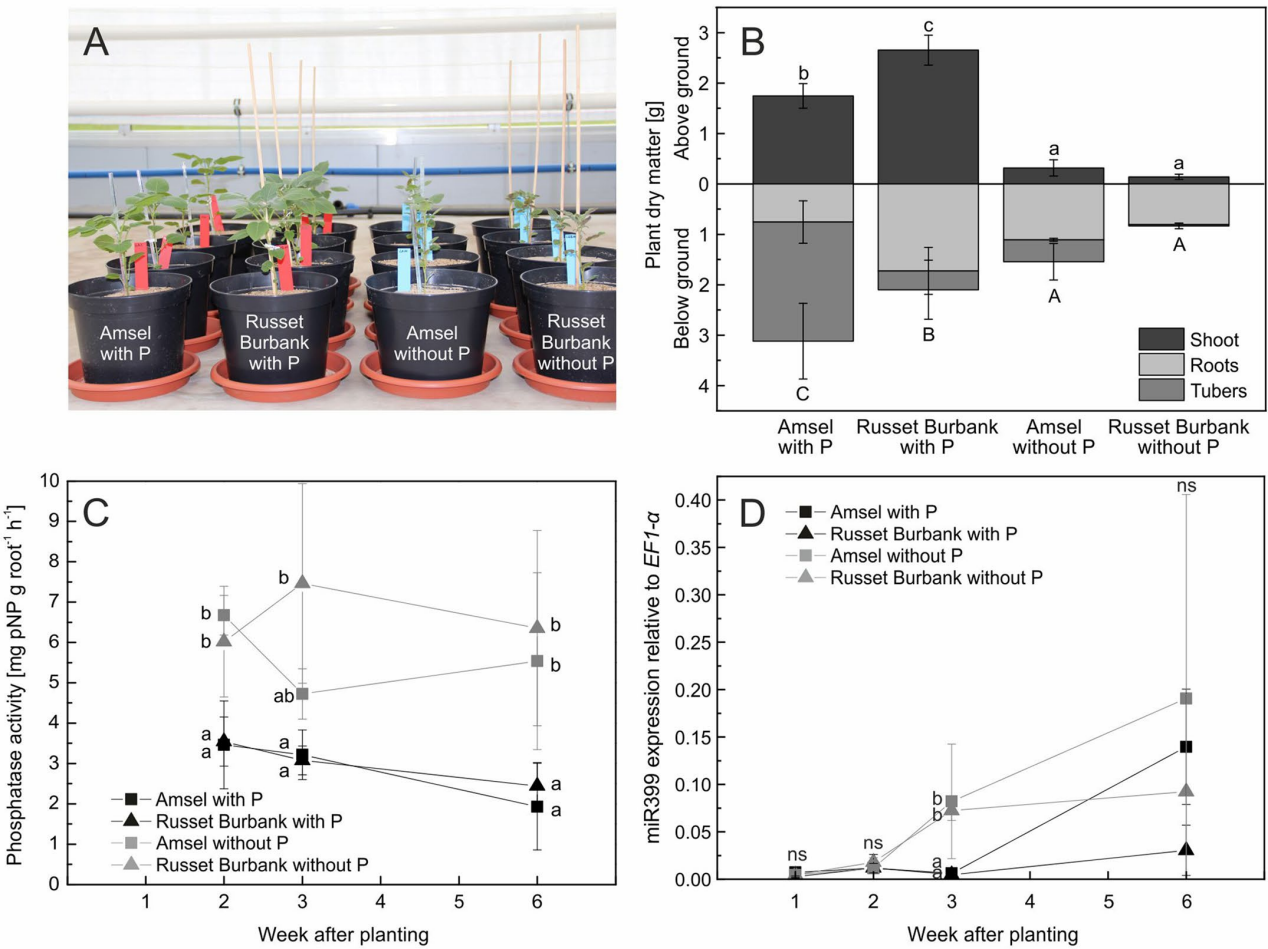
*In vitro* propagated potato genotypes grown in sand and fertigated with nutrient solutions with or without phosphate **(A)**, means and standard deviations of plant dry matter after 6 weeks of growth **(B)**, phosphatase activity on roots **(C)**, and miR399 expression relative to *EF1*-α expression **(D)**. Different letters indicate significant differences between genotype/treatment combinations **(B)** or treatments according to Tukey’s HSD [*p* < 0.05, *n* = four **(A**, **B**, **C)** or three **(D)** biological replicates].

Since plants increased rhizosphere acid phosphatase activity in Experiment B but different P treatments did not influence dry matter or phosphatase activity of plants raised from tubers, phosphatase activity was measured directly on roots of Amsel and Russet Burbank raised from *in vitro* propagated plants. Six weeks after planting, both cultivars were significantly smaller without P fertilization (*p* < 0.001, **Figure 5A**). Amsel had slightly higher dry matter and P content under no P than Russet Burbank (**Figure 5B**). Root phosphatase activity between plants grown with and without P differed significantly from the first sampling date 2 weeks after planting until the last sampling (*p* < 0.01), but was not different between the two genotypes (*p* > 0.05). The root phosphatase activity of plants with phosphate supply decreased from 2 to 6 weeks after planting from 3.5 mg pNP g^−1^ h^−1^ to 2.2 mg pNP g^−1^ h^−1^ while it was constantly at about 6 mg pNP g^−1^ h^−1^ for roots of plants without phosphate supply (**Figure 5C**).

After 1 and 2 weeks, miRNA miR399 expression in roots of plants grown both with P and without P was low, but differed significantly between the two P treatments after 3 weeks. The higher expression was measured in roots of plants grown without P (*p* < 0.001, **Figure 5D**). After 6 weeks, the expression of miR399 increased in roots of Amsel regardless of the treatment, while the expression level in Russet Burbank grown with P supply remained on a low level.

### Tuber Yields Under Field Conditions (Experiment D)

**Figure 6:**
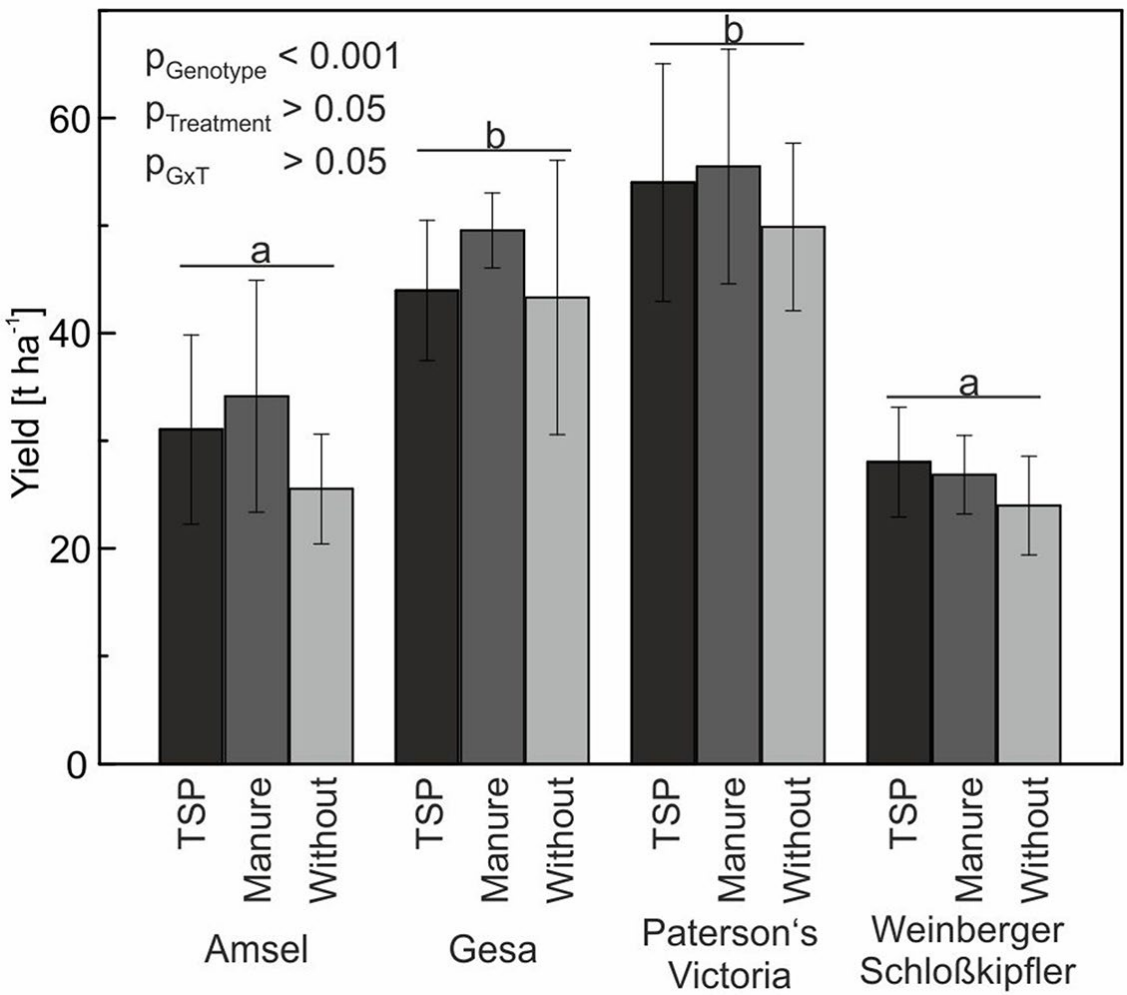
Means and standard deviations (*n* = four replicates) for tuber yield of potato cultivars in a long-term field experiment fertilized with triple superphosphate (TSP), manure or without phosphorus. Different letters indicate significant differences between cultivars according to Tukey’s HSD (*p* < 0.05).

Yield differences between the four genotypes Amsel, Gesa, Paterson’s Victoria, and Weinberger Schloßkipfler were statistically significant (*p* < 0.001). Highest yields were found for Paterson’s Victoria followed by Gesa, Amsel, and Weinberger Schloßkipfler. Although lowest tuber yields were observed for all genotypes in the no-P treatment and highest yields for all genotypes but Weinberger Schloßkipfler in the manure treatment, differences between the three P-fertilization strategies using TSP, manure, and no P were statistically not significant (**Figure 6**). Weather data for the growing period are given in **Supplemental Figure S3**.

## Discussion

### Genotypes Respond Differently to Soil-P Scarcity

Screening of 32 potato accessions in HP and LP (Experiment A) revealed that P content of potato genotypes cultivated under HP conditions was significantly higher than that of the genotypes cultivated under LP conditions (**Figure 1**). Significant genotypic differences were found for whole plant P content, PUtE, and dry matter in both HP and LP (**Figure 1**, **Table 1**, **Figure 2**), which supports earlier results from Sandaña (2016). Genotypic variation, although not significant, was found for responsiveness to P limitation, i.e., PAE (**Table 1**). PAE and PUtE contribute to P efficiency of plants and may differ between plant species and between genotypes within one species (Blair, 1993; Caradus, 1994). The correlation between PAE and relative dry matter indicates that PAE contributes to maintaining growth if soil P is the limiting factor. Although PAE was negatively correlated with PUtE in LP (**Figure 3**), PUtE seems not to have a strong effect on dry matter production. PUtE increased in LP of the present study but was not significantly correlated with dry matter in LP or relative dry matter production. The only significant correlation was observed between PUtE and dry matter in HP (**Figure 3**); hence, high biomass producers have small whole-plant P concentrations.

### Root System Size is Related to P Uptake

Optimal root growth is important for P efficiency. In P-deficient soils, the root/shoot ratio shifts towards the root (Raghothama, 1999; Schenk, 2006; van de Wiel et al., 2016). For field grown potatoes, it was shown that early root development correlated with P acquisition and tuber yield and the effect was stronger in low P soil (White et al., 2018). In our pot experiment (Experiment B), both root/shoot ratio and total root length did not differ significantly between P treatments (**Table 2**). Only specific root length increased significantly in LP, which gives a hint for an adaptation to soil P deficiency. However, since specific root length correlated negatively with whole plant P content (**Figure S2**), the increment is more likely a symptom of a low P status of plants. Although P treatments had no significant influence on total root length, a strong correlation between total root length and plant P content was obvious (**Figure 4**). The effect was stronger in HP and OP than in LP, where the correlation was not significant. Because of the low mobility of P in the soil, soil exploration by roots is one major strategy for plant P acquisition (Lynch, 2011). The P depletion zone around the roots reach usually not farther than a few millimeters and are even smaller in low P soil (Jungk and Claassen, 1989; Li et al., 1991). Hence, high correlations between root length and plant P content under properly fertilized conditions are not surprising. Especially in soils with low concentrations of plant available P, recycling P from the seed tuber might be more important. Accordingly, a high proportion of the accumulated P in potato was found to derive from the seed tuber in an earlier study (Pursglove and Sanders, 1981). It was also found for seedlings of soybean (Vandamme et al., 2016a) that seed weight and seed P content have an influence on plant growth at low P. Seed P is the main source for growth of maize in early growth stages (Nadeem et al., 2011).

The limited influence of low soil P contents on plant performance in Experiment B—probably due to tuber-P mobilization—is also reflected by the missing differences in dry matter production between treatments (**Table 2**). These were observed neither in the genotype screen, where growth duration under P scarcity was longer, nor in Experiment C, where *in vitro* propagated plants were used and the control plants were grown in sand without any P supply. In future studies, the influence of seed tuber P should be taken into consideration or excluded by using *in vitro* plantlets.

### Root Phosphatase Activity Increases Under P Deficiency

In this study, acid phosphatase activity in the rhizosphere was nearly three times higher than in control pots without plants (Experiment B, **Table 2**). Zimmermann et al. (2004) found that secreted phosphatase activity on potato roots increased under P deficiency. We found no statistical differences between treatments in Experiment B, when rhizosphere phosphatase activity was measured, but huge differences in root associated phosphatase activity measured directly on potato roots of Experiment C (**Figure 5C**). Nevertheless, plant P content in OP of Experiment B was as high as in HP (**Table 2**), which indicates P usage from phytate, although potato root phytase activity is very low compared to other crops (Zimmermann et al., 2003). Phosphate may have been released from phytate either by plants or by microorganisms present in the soil–sand mixture. Phytase activity secreted by roots is enhanced in P-deficient medium in many plants (Li et al., 1997). However, phytase activity of soil or roots was not measured. Wening (2016) found highly reduced growth and P concentrations in potato plants fertilized with Na-phytate compared to Ca(H_2_PO_4_)_2_ in a subsoil–sand mixture, but the same relation was not observed in a topsoil–sand mixture. Mineralization of phytate depended on both soil type and pH (Wening, 2016).

The two selected potato cultivars used in Experiment C displayed no differences in root phosphatase activity (**Figure 5C**). Above- and belowground dry matter production were statistically not different between cultivars without P (**Figure 5B**), and both cultivars had an intermediate PAE in the genotype screen, which might be the reason for similar behavior in root phosphatase activity. For maize, it was shown that low-P tolerant genotypes showed higher (Gaume et al., 2001; Jiang et al., 2017) or earlier (Du et al., 2016) root acid phosphatase activity than sensitive ones.

### Root Phosphatase Activity Was Not Controlled by miR399 Expression

In the present study, root-associated acid phosphatase activity was enhanced in plants grown without P before miR399 expression increased and did not further increase in relation to the increment of miR399 expression (Experiment C, **Figures 5C, D**). A strong local signal that occurred early during the experiment might have masked the later systemic signal mediated by miR399 expression, a key player in systemic P signaling (Chiou and Lin, 2011). In *Arabidopsis*, the activity of the major root-associated acid phosphatase AtPAP10 is mainly regulated by local signaling, i.e., P depletion on the root, and independent of the P status of the whole plant (Zhang et al., 2014). Transcription of *AtPAP10* already increased 2 h after transfer to a low P medium (Zhang et al., 2014). Transgenic tomato overexpressing miR399d from *Arabidopsis* had higher acid phosphatase activity in the root as well as secreted in low and high P solutions, compared to the wild type (Gao et al., 2010). This implies a dependency of root acid phosphatase activity on systemic P signaling in tomato.

### The P-Depleted Soil Did Not Reduce Tuber Yields Significantly

Soratto et al. (2015) emphasized the importance of classifying P responsiveness of potato cultivars’ in regard to tuber yield and not shoot or whole plant dry matter. Therefore, tuber yield of selected genotypes was evaluated in a field trial (Experiment D).

In the present study, tuber yield was slightly but not significantly reduced if plants were grown in a P-depleted soil and no P fertilizer was applied (**Figure 6**). According to Nawara et al. (2017), potato needs high soil-P concentrations to achieve 95% of optimal yield. Potato is assumed to be one of the more responsive crops to P fertilization. However, analyzing 197 data sets from long-term field trials in Germany and Austria, Buczko et al. (2018) found high variation in relative yield increase resulting from P fertilization. In our study, plant available soil P content did only differ slightly between control and TSP or manure treatment, although the control plots were not fertilized with P since 1998. These small differences suggest P translocation processes (Zicker et al., 2018) and mobilization from organic and/or inorganic sources in the soil, which might be the main reasons for small yield differences. In the same long-term field experiment and year as in our study, but not in other years, spring barley also showed no significant yield differences between organic and mineral fertilization strategies and zero-P fertilization control plots (Zicker et al., 2018). Since summer cereals are generally considered to be more susceptible to P deficiency than winter cereals and oilseed rape (Buczko et al., 2018; Zicker et al., 2018), results indicate that lowering the high P-fertilization level, which is common in Central Europe, may not directly reduce yield. However, Nyiraneza et al. (2017) found a high influence of year and site on yield and PUtE of field-grown potatoes; therefore, additional field trials are needed to verify our results.

## Conclusion

In the present study, we found differences in the shoot and tuber yields as well as in P contents between 32 potato cultivars under high and low P fertilizer treatments. Since P content of plants was related to total root length, we conclude that an exploratory and dense root system is one of the major goals in breeding for P efficient crops. Both root phosphatase activity and miR399 expression increased under P deficiency. Further studies with contrasting genotypes are needed to uncover the potential role of both traits to genetically improve P efficiency. In contrast to earlier studies in other plants, our results do not indicate that miR399 regulates root phosphatase activity in potato. Although potato is considered as one of the more susceptible crops to soil-P limitation and the genotype screen resulted in differences in dry matter production, tuber yield of potato cultivars grown on plots with suboptimal contents of available P was not significantly reduced in comparison to well-fertilized plots. In conclusion, lowering P fertilizing recommendations might not necessarily reduce tuber yields.

## Data Availability

All data sets are available from the corresponding author on reasonable request.

## Author Contributions

KW-F, MK, and VP conducted experiments; KW-F, MK, and RU analyzed data; RU and KD designed the experiments; KW-F, MK, RU, KD, VP, and SB-P wrote the manuscript. All authors contributed to manuscript revision and read and approved the submitted version.

## Funding

This research was funded by the Leibniz Association within the scope of the Leibniz-ScienceCampus Phosphorus Research Rostock (www.sciencecampus-rostock.de). We acknowledge financial support by Deutsche Forschungsgemeinschaft and Universität Rostock within the funding programme Open Access Publishing.

## Conflict of Interest Statement

The authors declare that the research was conducted in the absence of any commercial or financial relationships that could be construed as a potential conflict of interest.
