# Supplementary material for: Genotype-Specific Differences in Phosphorus Efficiency of Potato (Solanum tuberosum L.)
Source: Front Plant Sci. 2019 Aug 16;10:1029. doi: 10.3389/fpls.2019.01029 (PMC6706458; doi:10.3389/fpls.2019.01029)
Supplement: Supplementary file 1 [file DataSheet_1.pdf]

Supplementary Material

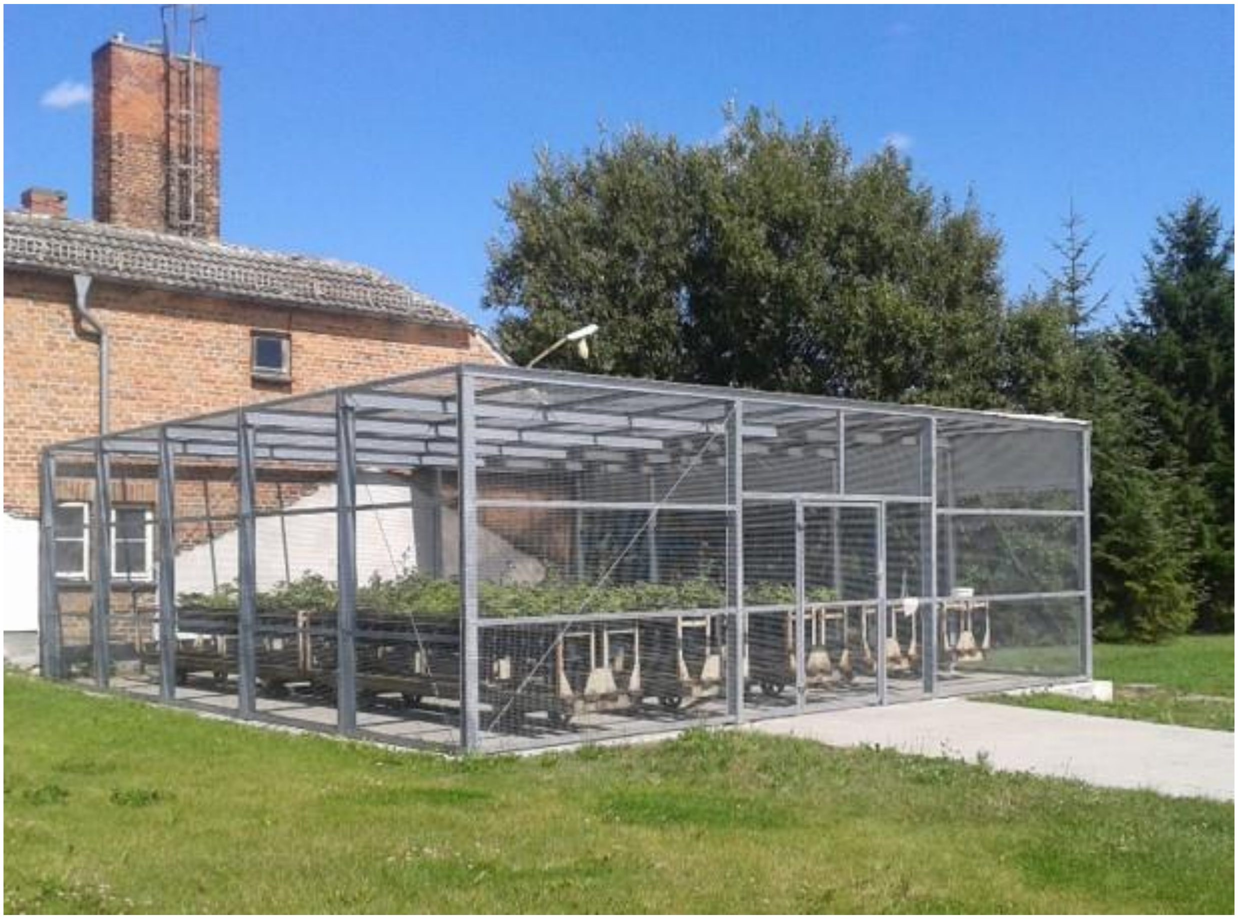

Supplementary Figure S1. Large wire cage used in Experiment A.

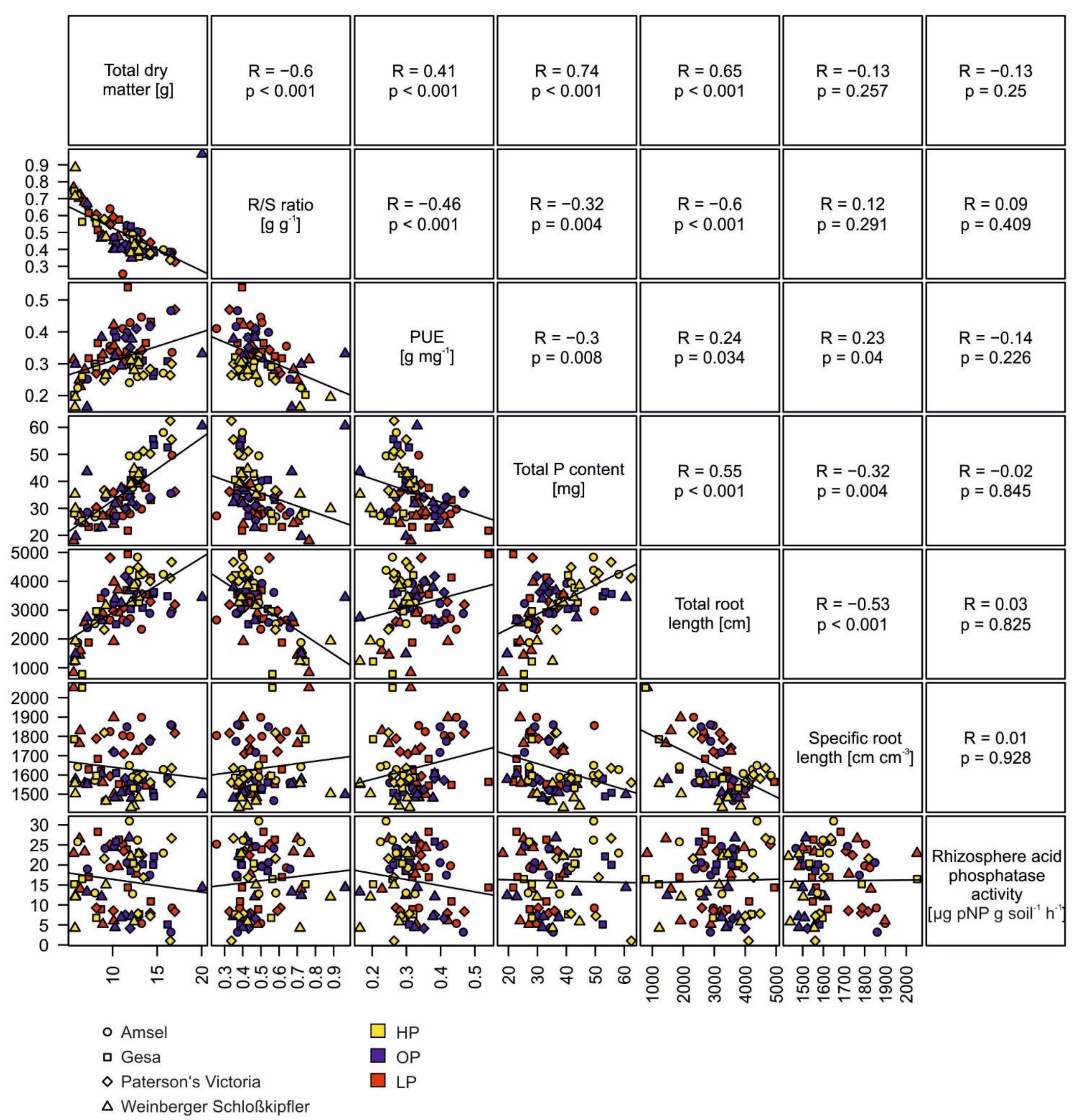

Supplementary Figure S2. Pearson's correlation coefficients between plant parameters of the potato cultivars Amsel (circle), Gesa (square), Paterson's Victoria (diamond) and Weinberger Schloßkipfler (triangle) grown in pots fertilized with KH_2PO_4 (yellow), $C_6H_{16}CaO_{24}P_6$ (blue) and without P supply (red).

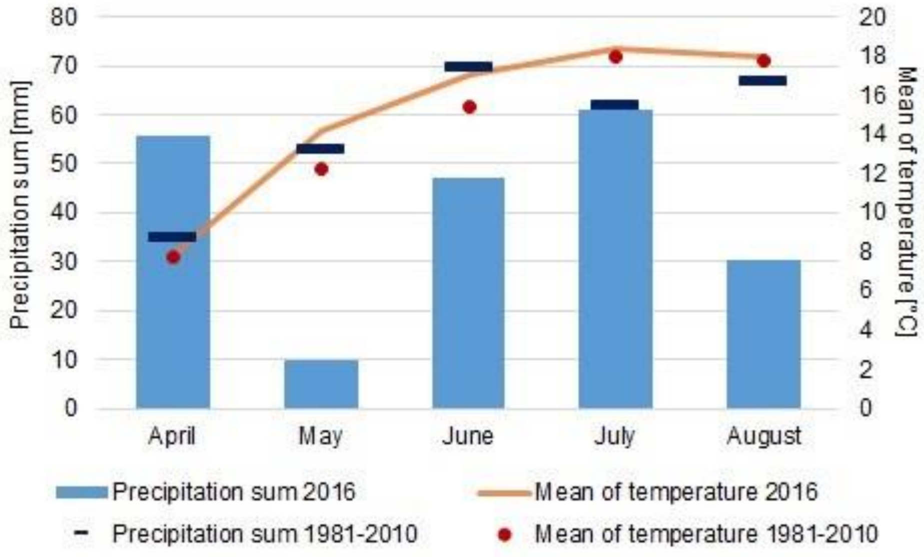

Supplementary Figure S3. Monthly precipitation sum and temperature for growing period 2016 (calculated from values per day) and mean of 1981-2010 (data from Deutscher Wetterdienst, www.dwd.de) for the nearest weather station in Rostock-Warnemünde.
